# “Intergenerational and Intra-generational Equity Under the BBNJ Agreement; Advancing Accountability Towards Sustainable Management of the Marine Environment”

**DOI:** 10.1007/s00267-025-02256-5

**Published:** 2025-08-23

**Authors:** Zakieh Taghizadeh

**Affiliations:** The Institute for International Business Law, University of Fribourg, Fribourg, Switzerland

**Keywords:** Marine Biological Diversity, Intergenerational Equity, Intra-generational Equity, BBNJ Agreement, Indigenous Ecological Knowledge, Intergenerational Responsibility, Marine Environmental Management

## Abstract

Intergenerational and intra-generational equity have gained increasing significance in the development of international environmental law, particularly in response to the accelerating loss of marine biodiversity. The landmark Agreement on the Conservation and Sustainable Use of Marine Biological Diversity of Areas Beyond National Jurisdiction (BBNJ Agreement) introduces a novel legal framework for global ocean governance, recognizing the shared responsibility of States to manage and sustainably use marine biological diversity for both present and future generations. This article examines how the BBNJ Agreement incorporates and operationalizes sustainable equity principles and assesses the implications of the inter-/intra-generational principles for advancing environmental management across theory, policy, and practice. Specifically, it explores how the common heritage of humankind principle and the precautionary approach can inform an integrated, equitable system for managing marine genetic resources (MGRs) as global commons resources in areas beyond national jurisdiction. The analysis highlights pathways for embedding accountability and stewardship in international marine policy towards future generations, and offers a framework for balancing inter-/intra-generational equity asymmetries in decision and policy-making processes. By bridging legal principles with environmental management strategies, the article contributes to more inclusive, responsible, and forward-looking stewardship of marine biological diversity in areas beyond national jurisdiction.

## Introduction

In recent years, the international community has grown increasingly aware of the pressing need to confront global environmental challenges to ensure the sustainability of our shared planet for both present and future generations. International legal mechanisms have played a crucial role in shaping environmental norms and guiding the equitable use of shared natural resources for the sustainable development of all societies.^1^ The Agreement on the Conservation and Sustainable Use of Marine Biological Diversity of Areas Beyond National Jurisdiction (the BBNJ Agreement) as an implementing agreement under the United Nations Convention on the Law of the Sea (UNCLOS), represents a landmark development in the governance of marine biodiversity in areas beyond national jurisdiction (ABNJ), including its ecosystems and resources. It establishes a comprehensive legal framework to promote the conservation and sustainable use of marine biological diversity in areas beyond national jurisdiction, grounded in principles such as the common heritage of mankind/humankind 2 and the precautionary approach 3.

Through explicitly acknowledging the need to act as “stewards of the ocean on behalf of present and future generations,” the BBNJ Agreement recognizes the importance of long-term environmental responsibility and sustainable management of the marine biological environment along with its resources (De Lucia 2024). It recognizes the complex interdependence between human activities and marine ecosystems and underscores the need for holistic, integrated approaches to environmental management that address both ecological integrity and intergenerational responsibility. Nevertheless, the question remains surrounding what constitutes legal components of accountability towards future generations concerning marine biodiversity conservation and environmental biological management.

To address these questions, the following article critically examines the concepts of inter-/intra-generational equity within the framework of international law, with a particular focus on their articulation and implications for environmental governance under the BBNJ Agreement. Intergenerational equity refers to the principle of fairness and justice in allocating environmental resources and their enormous benefits across generations. It requires a reconceptualization of the concept of sovereignty and legal obligations, positioning the present generation as both beneficiaries and trustees of Earth’s natural legacy (Burch 2019; Collins 2007). It recognizes that current actions and decisions have lasting impacts on the well-being of future generations, thereby necessitating a balanced approach that takes into account the needs and priorities of all stakeholders, both present and future generations. Conversely, intra-generational equity focuses on addressing fairness and justice within the present generation, emphasizing the need for inclusivity, access to social justice, and shared accountability, especially in the context of global disparities and injustices (Sulyok 2024; Foster 2024).

The central research question in this article revolves around: How does the BBNJ Agreement implicitly contribute to the development of intergenerational equity as a legal norm and practical framework for advancing marine biodiversity governance and sustainable management of marine resources? To answer this question, first and foremost, the article explores how the Agreement reflects intergenerational equity paradigms in its normative structure. It will then identify specific legal and institutional mechanisms through which inter-/intra-generational equity might be operationalized, and finally propose targeted policy and governance recommendations for embedding these principles into future BBNJ implementation strategies.

This article situates the BBNJ Agreement within broader theoretical discourses on equity in environmental law and marine biological governance, to advance the growing literature on transboundary environmental accountability and the international community’s evolving role as collective agent and responsible “steward” of the global ecological commons. Beyond contributing to legal theory, it addresses a pressing implementation gap by proposing concrete accountability mechanisms and participatory frameworks to embed inter-/intra-generational equity within the BBNJ Agreement’s operational framework. These include transparency-enhancing provisions and measures, intergenerational impact assessments, and the adaptation of public trust doctrines to the unique legal context of areas beyond national jurisdiction. By advancing these frameworks, the article advocates for the elevation of intergenerational equity from rhetorical aspiration to enforceable legal standard, positioning the BBNJ Agreement as a timely normative framework for promoting a more just, participatory and forward-oriented model of global environmental governance that affirms the shared responsibility of the international community to safeguard the planet's ecological heritage for both present and future generations.

## The International Legal Framework Surrounding Intergenerational Equity: Specific Examination of the BBNJ Agreement

The principle of equity, particularly when framed through intergenerational and intra-generational dimensions, has become central to contemporary debates on global environmental governance, marine biodiversity conservation, and benefit-sharing arrangements arising out of common shared resources. This section critically examines the evolving international legal landscape concerning inter-/intra-generational equity, focusing specifically on how the BBNJ Agreement advances or fails to operationalize this normative commitment. The study will delve into the broader international legal framework that seeks to address inter-/intra-generational equity, highlighting the shifts from traditional concepts of resource governance towards more inclusive and future-oriented approaches that strive to minimize potential harm to future generations. Ultimately, the second part of this section shifts focus specifically to the BBNJ Agreement, critically examining its provisions for ensuring equity and the Agreement’s potential to balance the interests of present and future generations.

### Transformative International Legal Framework Regarding Equity Concepts

The notion of intergenerational equity as a futuristic principle of international environmental law (Weiss 2021), advocates for the rights of future generations to inherit an ecologically viable planet, while intra-generational equity concerns the fair distribution of environmental benefits and burdens within the current generation. These dual components form the foundation of the sustainable equity paradigm, which is increasingly recognized as a transformative principle in modern international law. Far from remaining aspirational, these concepts are operationalized across various international legal frameworks and judicial decisions that aim to address the challenges of sustainability and equity. For instance, Article 3.1 of the United Nations Framework Convention on Climate Change (UNFCCC) calls for the protection of the climate system “on the basis of equity” for both present and future generations. This section of the article will analyze how evolving norms and emerging legal instruments integrate the principles of inter-/intra-generational equity into the governance of the global commons and particular issues, including but not limited to biodiversity conservation, climate change, and marine genetic resources. Rather than approaching intergenerational equity as an abstract or rhetorical notion, the article focuses on its concrete legal manifestations, with attention to how recent legal instruments seek to integrate future-oriented approaches into resource governance structures.

#### Defining Future Generations’ Concerns in Equity-Based International Law?

International environmental law has traditionally focused on the rights and obligations between states within the present generation, often overlooking the long-term ecological impacts of present decisions on future generations. However, the intensifying challenges of climate change, biodiversity loss, and high seas degradation have catalyzed an evolving recognition that justice must extend beyond temporal and intergenerational boundaries.

Within this evolving paradigm, sustainable equity principles provide a critical entry point for this temporal perspective, calling for the incorporation of future-oriented criteria into the procedural and substantive obligations of states under international law.^4^ The principle of sustainable equity is multi-dimensional: it functions as a legal principle, an interpretative tool in treaty implementation, a mechanism for dispute resolution, and a normative foundation for emerging customary rules and practices (Francioni 2024). This is exemplified in international legal obligations, such as the equitable and reasonable use of shared watercourses and natural resources (*UN Convention on the Law of the Non-Navigational Uses of International Watercourses*, 1997, Article 5) and the duty to safeguard the climate system for the benefit of both present and future generations (Gottlieb. et al. 2025).

At the heart of the sustainable equity paradigm lies the most privileged legal premises arising from the principles of inter-/intra-generational equity, with their “ancient roots in moral philosophy of justice and traditional legal systems” (Pedersen and Sulyok, 2024, 5). While the principle of intra-generational equity addresses inequalities and disparities among states and communities within the present generation, reflected in mechanisms such as benefit-sharing and technology transfer (Dasgupta and Mitra 1983), intergenerational equity mandates that legitimate interests of current populations do not override the ecological entitlements of future generations to access marine biodiversity, benefit from marine genetic resources, or exercise environmental rights (Dasgupta and Mitra 1983).

The inter-/intra-generational equity concepts collectively reflect the inter-temporal and trans-temporal dimensions of natural resources governance, extending the temporal aspect of well-established obligations under international environmental law (Pedersen and Sulyok 2024). These evolving principles recognize the rights and obligations of the current and future generations regarding the sustainable use of the natural common resources of humanity with due regard to the importance of maintaining the proper temporal balance of the environment. This approach guarantees a comprehensive balance between distributive justice and conservation approaches to marine genetic resources while serving the legitimate interests of present and future generations. (Taghizadeh 2024).

The two equity-based principles shape a holistic approach towards management of the global commons, particularly in domains such as the marine environment, where resource exploitation poses long-term environmental, social, and ethical implications. These principles are increasingly codified through recent international instruments and soft law frameworks, such as the UN Sustainable Development Goals, (e.g., SDG10, which refers to reducing inequality within and among countries, and SDG 14) and global initiatives like the UN Decade of Ocean Science (Ocean Decade, 2023). This progressive integration of equity enables historically marginalized and underrepresented groups to move from passive beneficiaries to achieve “driving seat” in initiating partnerships and setting priorities for capacity development in ocean governance, demanding a deeper understanding of the meanings, motivations, pathways, and measurements of equity (Harden-Davies et al. 2023).

Given the intensifying environmental crises of the Anthropocene, international legal and policy frameworks must undergo a ‘deep, sustained, and systemic’ transformative shift towards securing the long-term sustainability and resilience of ocean ecosystems and their resources (Linnér and Wibeck 2020). Equity in this context must be reimagined not merely as an abstract altruistic value but as an operational principle embedded in legal structures and governance mechanisms to evaluate how benefits, burdens, and responsibilities of ocean use are equitably distributed within and across generations. Realizing this transformation requires moving beyond rhetorical commitments to equity and embedding the inter-/intra-generational equity in the procedural and institutional fabric of global environmental governance.

#### The Inclusion of Future Generations and Equity Concepts into the International Legal Framework: From the 1972 Stockholm Declaration to UNCLOS as the Constitution for the Oceans

Arguably, the legal cradle of future generations' concerns is international law, and its principles of sustainable development and sustainable equity paradigms (Pedersen and Sulyok 2024). The sustainable equity and future generations concepts have found explicit consideration in international legal frameworks, forming part of international and domestic laws for decades, whether in the form of perambulatory provisions or as a background principle (Weiss 1989). The concept of intergenerational and sustainable equity is particularly relevant in the context of the high seas and the Area, which are largely governed by international agreements and mechanisms.

The United Nations Framework Convention on Climate Change (UNFCCC) (Art. 3) and the 2015 Paris Agreement^5^, the United Nations Convention on the Law of the Sea (Arts. 192, 117 and 118), the Convention on Biological Diversity (CBD preamble, Art. 2), the World Heritage Convention (Art. 4), and the United Nations Economic Commission for Europe Convention on the Protection and Use of Transboundary Watercourses and International Lakes (also known as Water Convention) contain explicit references to the obligations towards future generations. The principle is also mentioned, *inter alia*, in other soft law instruments such as the 1972 Stockholm Declaration (principles 1 and 2), and the Rio Declaration (principle 3) (Rawls 1971). The Convention on Biological Diversity (CBD), inter alia, incorporates the principle of intergenerational equity in its objective on sustainable use of components of biodiversity, defining “sustainable use” to mean the use of components of biological diversity in a manner that preserves the “potential to meet the needs and aspirations of present and future generations” (Arts. 1 and 2 of the CBD).

Apart from the CBD, the concept of future generations has been embedded in various provisions of the UNCLOS, which establishes a comprehensive framework for the rights and responsibilities of states in the use and management of the oceans. UNCLOS obliges present generations to take active steps to protect and preserve the marine environment for the benefit of humankind, as highlighted by Article 192 (UNCLOS, Part XII). This general duty sets the stage for specific provisions that address ABNJ, including the high seas and the Area (the seabed and ocean floor beyond national jurisdiction), in ways that directly impact future generations.

The UNCLOS framework for ABNJ has significant implications for the rights and interests of future generations. Notably, Articles 117 and 118 underscore the importance of international cooperation and taking appropriate measures in managing the high seas and conservation of its living resources that fall outside the jurisdiction of any state. In the absence of such cooperation, these areas risk becoming a “tragedy of the commons” where unregulated exploitation leads to resource depletion and environmental degradation. By prioritizing conservation and collaborative management, these provisions reflect core principles of sustainable development, echoing broader commitments such as those found in the Rio Declaration, which urges environmental protection to “equitably meet the developmental and environmental needs of present and future generations.” (Rio Declaration, Principle 3).

Beyond the high seas, UNCLOS addresses the Area through the ground-breaking principle of the “common heritage of mankind” (CHM), as one of the most important achievements of UNCLOS enshrined in its Part XI. The CHM principle designates the Area and its resources as the shared heritage of all humankind, explicitly including future generations as beneficiaries and an inalienable part of ‘mankind’ or rather ‘humankind’ (Ivanova 2023). This approach would inherently incorporate intergenerational equity by viewing the Area’s resources as a legacy to be preserved, not only for current use but also for future generations. Such interpretation aligns with scholarly discussions on ocean governance, which explores how UNCLOS integrates intergenerational equity into its framework for managing marine resources (Fitzmaurice 2018; Wouters 2013).

The Convention thus establishes the International Seabed Authority (ISA) to act as a steward of the Area, and a ‘trustee’ on behalf of humankind, ensuring that activities in the Area, such as deep-sea mining, are conducted in a manner that benefits all humankind and protects the marine environment. (Ivanova 2023, 339). Article 145, for example, mandates the ISA to take measures to “ensure effective protection for the marine environment from harmful effects which may arise from [activities in the Area]” (UNCLOS, Part XI, Article 145). This includes conducting environmental impact assessments and enforcing due diligence, as emphasized in the Advisory Opinion by the International Tribunal for the Law of the Sea (ITLOS) Seabed Disputes Chamber, which underscores the obligation to protect the marine environment for future generations (Kittichaisaree, 2025).

### BBNJ Overarching Principles and Innovative Mechanisms Relating to Inter-/intra-Generational Equity

The international legal framework for inter-/intra-generational equity has been shaped by various mechanisms and overarching principles that recognize the importance of effective conservation and sustainable use of marine biodiversity in a manner that takes into account the needs of both present and future generations. The overarching guiding principles under the BBNJ agreement, ranging from equity, equitable sharing of benefits, and sustainable development to the “precautionary approach or principle”^6^ “polluter pays” and the common heritage of humankind principle (CHP) reflect a commitment to inter-/intra-generational equity, striving to protect and preserve our shared oceans and common resources for future generations (Gottlieb. et al. 2025).

From a methodological perspective, the BBNJ Agreement provides a compelling illustration of how inter-/intra-generational equity can be translated from a normative principle into institutional practice. To this end, the Agreement creates procedural and substantive opportunities for integrating equity into environmental management, particularly through measures such as intergenerational impact assessments, participatory governance mechanisms, and transparency obligations. This part of the article will explore these overarching principles and innovative mechanisms that underpin inter-/intra-generational equity within the BBNJ Agreement. Through this examination, the study aims to evaluate the BBNJ Agreement’s capacity to contribute to a more just and sustainable international legal order, capable of addressing the complex challenges surrounding environmental degradation facing current and future generations.

#### Legal Analysis of the Guiding Principles Relating to Inter-/intra-Generational Equity

##### Under the BBNJ Agreement

The principles of sustainable equity, the precautionary approach, the common heritage of humankind principle (CHP), and most significantly, inter-/intra-generational equity are among the essential components of international environmental law that aim to protect the environment and promote equity and fairness for future generations. These principles intersect in their emphasis on responsible decision-making and stewardship of the global commons, emphasizing the importance of taking preventive measures and considering the long-term impacts of human activities on the environment. The following section of the article will explore the connections between the precautionary principle, intergenerational equity, and the CHP, highlighting their importance in promoting sustainable practices and equitable resource management.

##### The Precautionary Principle and Intergenerational Equity

International environmental law requires the application of preventive measures, most notably the precautionary principle, to safeguard transboundary and global ecosystems, especially where there is a risk of serious harm to human health or irreversible damage to the ecological environment.^7^ The precautionary principle is a key governance pillar of international law based on “common sense notions of harm avoidance” (Dunn 2021), urging states to “apply precaution” even in the face of scientific uncertainty (Sandin 1999; Gardiner 2006). Enshrined in international agreements such as the Rio Declaration on Environment and Development,^8^ it states that “where there are threats of serious or irreversible damage, lack of full scientific certainty shall not be used as a reason for postponing cost-effective measures to prevent environmental degradation”. This principle promotes equity in international law by requiring states to take precautionary measures to minimize potential harm to the environment, even in the absence of definitive proof of harm.

The precautionary principle is particularly significant in the context of the high seas and the Area, where the long-term impacts of human activities may cause significant irretrievable harm to the shared environment (Gardiner 2006, 33-60). According to the implications arising out of the precautionary approach, the burden of proof lies with the state or private individuals to substantiate that their development activities are thoroughly sustainable and do not cause harm to the environment and global commons.^9^Hence, to prevent irreversible harm to the global commons, particularly marine resources, it is imperative to foresee a comprehensive international responsibility regime entailing a precautionary approach that guarantees a proper determination of regulations and laws in the field of international environmental law and global commons protection. Viewed through a rigorous scientific lens, the precautionary principle is not simply a statement of intent but a practical tool embedded in the BBNJ Agreement’s environmental impact assessment (EIAs) framework to ensure proactive, adaptive governance of marine biodiversity beyond national jurisdiction. Its role transcends mere rhetoric; it operationalizes environmental protection where scientific ambiguity exists (Lothian 2022).

Despite its prominence in treaties like the UN Framework Convention on Climate Change (1992) and the Cartagena Protocol on Biosafety (2000), the precautionary principle’s normative status as a customary international law principle remains under debate. Proponents argue that the principle’s widespread inclusion in international agreements and national laws suggests an emerging customary norm. However, customary international law requires consistent state practice and *opinio juris* (acceptance as legally binding). International courts, such as the International Court of Justice (ICJ) in cases like Pulp Mills (ICJ Judgment, Pulp Mills Case 2010) and Gabčíkovo-Nagymaros (ICJ Judgment, Gabčíkovo-Nagymaros Case 1997), have referenced the principle but have not definitively recognized it as customary international law, often citing insufficient evidence of uniform state practice or *opinio juris*. Likewise, the International Tribunal for the Law of the Sea (ITLOS) in the Southern Bluefin Tuna Cases (1999) remained silent on explicitly recognizing the precautionary approach as a customary norm, yet applied it implicitly in its decision (ITLOS Southern Bluefin Tuna Cases, 1999).

The precautionary approach and futuristic principles of environmental sustainability law are intertwined in a way that contains significant elements regarding future-oriented, transformative, and inter-temporal obligations towards posterity (Tladi 2007). From this perspective, the precautionary principle and inter-/intra-generational equity are two sides of the same coin, as both seek to protect the interests and well-being of current and future generations by promoting responsible and sustainable decision and policy-making processes as well as boosting public-private partnerships (Castaldo et al. 2025). A precautionary approach towards preventing harm to the marine environment also ensures that future generations will inherit a healthy and thriving environment and promote inter-/intra-generational equity and fairness towards resource management in a forward-looking and future-oriented manner (Page 2012).

An explicit illustration of the interaction between the precautionary principle and intergenerational equity in practice includes the regulation surrounding the introduction of new gene technologies into the ecosystem, particularly genetically modified organisms (GMOs).^10^ This is now more evident than ever that GMOs can substantially affect both human health and the environment, yet there is considerable uncertainty regarding their long-term effects (Noack et al. 2024). Therefore, the precautionary principle advocates for a cautious approach, necessitating strict regulations to mitigate potential harms (Cooney 2004). While the concept of intergenerational equity, on the other hand, emphasizes the importance of evaluating the consequences of GMOs for future generations, ensuring that they are not adversely impacted by detrimental human actions (Noack et al. 2024).

##### The Common Heritage of Mankind Principle and Inter-/intra-Generational Equity

The common heritage of mankind (CHM), which is viewed as a cornerstone of international law (Owolabi 2013; Wang 2021), embraces a holistic framework towards shared global commons stewardship and sustainable management. Originating in the 1967 proposal by Maltese Ambassador Arvid Pardo for the deep seabed and codified in UNCLOS (Part XI, Article 136), the CHM principle posits that certain global commons including areas or resources beyond national sovereignty of states are the shared inheritance of all humanity, to be preserved collectively through the international community ensuring an equitable distribution of benefits among present and future generations as beneficiaries of the ocean’s biological diversity (Wolfrum 2009).

The CHM principle comprises five key components of non-appropriation (no state or entity can claim sovereignty over the resource) (Article 137 of UNCLOS), international management (An international authority oversees the resource’s use), (Part XI of UNCLOS) benefit-sharing (benefits, whether monetary or non-monetary, are shared equitably among states) (Article 140 of UNCLOS), peaceful use (the resource is used for peaceful purposes only) (Article 141 of UNCLOS) and conservation for future generations (the resource is preserved for future use, ensuring sustainability). These components underpin the principle’s role in promoting intra-generational equity (fair access among current states, particularly for developing countries) and intergenerational equity (preserving resources for future generations) (Principle 2 of the Stockholm Declaration, and Principle 3 of the Rio Declaration). As scholars have noted, the dual invocation of “humanity” and “heritage” in the CHM underscores a legal and moral obligation to balance present needs with future rights, fostering responsible stewardship of the global commons (Dolzer, et al. (Eds.) 1989). The CHMthus becomes a site where structural inequalities in global environmental governance are both reproduced and resisted. (Vadrot et al. 2022).

Although the CHM principle enjoys customary international law status in the context of the deep seabed, as confirmed by consistent state practice, *opinio juris* (ICJ Statute, Article 38(1)(b)) and widespread implementation through the ISA, its legal applicability to marine genetic resources (MGRs) in areas beyond ABNJ, particularly in the high seas water column, remains legally contested. Developing countries (mostly orchestrated through the Group of 77+China) have long advocated for the CHM to be part of customary international law, ensuring equitable access to its benefits for the whole international community, particularly for countries lacking technological capacity to exploit these common resources. Scholars also argue that UNCLOS’s near-universal ratification supports the CHM’s customary status for seabed minerals (Becker and Derrig 2023). Nevertheless, resisted opposition from developed states invoking the principle of freedom of the high seas and marine scientific research coupled with the absence of a definitive adjudication by an international court, has contributed to the principle’s ambiguous status beyond the seabed and within the high seas water column.^11^

To avoid these ideological disagreements, the BBNJ Agreement negotiations, after intensive debates over a decade, strategically adopted a pragmatic approach, focusing on benefit-sharing mechanisms rather than fully resolving MGRs’ legal status under the CHM doctrine (Mendenhall et al. 2023). Subsequently, the final text of the BBNJ Agreement reinstates the common heritage of humankind as a guiding principle in Article 7(b), alongside benefit-sharing provisions, as outlined in Part II of the BBNJ Agreement.^12^ The resulting compromise applies the language of “benefit of all humanity” in the context of MGRs, incorporating the common heritage principle alongside other guiding principles to govern the legal framework while establishing a tailored benefit-sharing regime and associated digital sequence information (Kachelriess 2023).

The inclusion of the common heritage of humankind as a fundamental principle in the BBNJ Agreement elevates inter-/intra-generational equity from abstract values to legal imperatives and marks a significant achievement in the progressive development of the law of the sea (Taghizadeh 2024). This integration strengthens the Agreement’s normative weight towards a commitment to collective stewardship of marine common resources, reinforcing significant support for the view that the principle of common heritage of humankind, alongside its well-established components, forms a normative foundation for both inter-/intra-generational equity, and this approach should extend well beyond mere benefit-sharing to incorporate both distributive and conservation-oriented obligations (Taghizadeh 2024).

Moving beyond, a clear and robust interpretation of the CHM/CHP in consistency with its constituent elements and legal status is essential to securing the international community’s commitment to sustainable equity, responsible stewardship of marine biodiversity, and the promotion of transparency, accountability, and global justice for future generations. Policymakers and stakeholders must strive to uphold the core legal values and ethical elements enshrined in the common heritage of humankind framework, to realize its full potential in promoting responsible, equitable, and sustainable utilization of marine genetic resources for the benefit of all generations, particularly for vulnerable states and indigenous peoples, and local communities. Ultimately, the inclusion of the common heritage principle obliges Parties to design benefit-sharing and governance mechanisms that frame MGR utilization in the benefit of all humanity, integrate conservation-focused, long-term safeguards, ensure transparency, accountability, and participatory decision-making, and provide capacity-building for equitable global participation (Humphries. Ed. 2025).

#### The Evolutionary BBNJ Mechanisms to Ensure Sustainability and Protection of Marine Biodiversity for Future Generations

The inclusion of specific provisions in the BBNJ Agreement further demonstrates a profound commitment to sustainable equity, notably inter-/intra-generational equity, by introducing robust mechanisms designed to protect and preserve marine biodiversity for the benefit of all humankind. Several of these mechanisms and tools are uniquely pioneering, either by providing new safeguards for the marine environment or by advancing the conservation instruments found within existing biodiversity frameworks. These mechanisms include progressive developments in the conservation and sustainable use of marine biodiversity, such as the establishment of area-based management tools (ABMTs), the normative development of environmental impact assessment frameworks, and the introduction of new measures to address the impacts of human activities on marine biodiversity in ABNJ.

One of the evolutionary aspects of the BBNJ Agreement is its requirement specifying that all emergency measures, review processes, and monitoring activities be grounded in the *best available science and scientific information* and, where available, *traditional knowledge of Indigenous Peoples and local communities*, taking into account the *precautionary approach* and an *ecosystem approach*. (Article 26 of the BBN Agreement). The application of these principles and approaches ensures that the BBNJ Agreement fulfils its objective towards protecting and preserving marine biodiversity, particularly the legal status of global commons, for the benefit of both present and future generations as the potential beneficiaries of the Agreement.^13^

Significantly, Part II of the Agreement established a comprehensive and equitable benefit-sharing regime concerning monetary and non-monetary benefits derived from MGRs, including digital sequence information (DSI), the transfer of marine technology, and capacity-building activities (Part II of the BBNJ Agreement). Central to the negotiation process was the debate over whether such transfers and capacity-building measures should be mandatory or remain voluntary, and whether benefit-sharing should entail financial obligations for participating states (Tiller et al. 2019). The final framework emphasizes fair and equitable distribution of benefits across the entire international community, with special consideration for the unique circumstances of developing and least-developed countries as well as Small Island Developing States (SIDS), thereby reinforcing the normative commitment to distributive justice in the governance of ocean commons (Gottlieb. et al. 2025).

To operationalize these objectives, the Agreement introduces several innovative institutional mechanisms, such as an Access and Benefit-Sharing Committee and a digital open-access platform– Clearing-House Mechanism to facilitate transparent access to MGR-associated data and to promote scientific collaboration (BBNJ Agreement, Article 15 and 51). Furthermore, the agreement includes a notification system and the use of batch identifiers to enable traceability and accountability across various stages of the MGR value chain and support compliance (Oldham et al. 2023). The Agreement also stipulates that monetary benefits must be deposited into a “special fund” that will support the implementation of assistance and capacity-building activities, ensuring that all benefits shared from MGR-related activities will be directed towards the objectives of the BBNJ Agreement.^14^ While the exact modalities for sharing monetary benefits are yet to be determined by the BBNJ Conference of the Parties (COP), such a framework nonetheless represents a significant move toward equity and sustainability in the governance of MGRs, rooted in the overarching principle of the CHP.

Additionally, Part III of the Agreement outlines the establishment of a comprehensive system of area-based management tools, including ecologically representative and well-connected networks of marine protected areas. These tools are intended to conserve and sustainably use marine biodiversity and protect vulnerable marine ecosystems, implicitly with a view to ensuring the well-being of future generations (Article 17 of the BBNJ Agreement). It further specifies that the establishment of ABMT explicitly aims to “protect, preserve, restore and maintain biological diversity and ecosystems, including to enhance their productivity and health, and strengthen resilience to stressors, including those related to climate change, ocean acidification and marine pollution.”

Part IV of the Agreement, *inter alia*, introduces a structured mechanism for conducting Environmental Impact Assessment (EIAs) and other appropriate requirements for reporting assessments by Parties of activities in ABNJ. Article 1(7) defines EIAs as processes that identify and evaluate the potential impacts of an activity to inform decision-making. Article 28 of the Agreement lays down an obligation for states to conduct EIAs for planned activities under their jurisdiction or control that may have potential impacts on the marine environment, thereby reinforcing the obligation to act responsibly within the international commons.

The BBNJ agreement also provides for capacity-building and technology transfer to support the conservation and sustainable use of marine biodiversity, particularly in developing countries, with due regard to the least-developed countries and particularly SIDS. (Part V of the BBNJ agreement) The objective is to develop marine scientific and technological capacity, enhance access to relevant technologies, and promote cooperation and knowledge-sharing regarding biodiversity conservation in ABNJ. To achieve these objectives, Article 41 of the BBNJ agreement underscores the importance of international cooperation in the transfer of marine technology and capacity-building activities, obliging developed countries to provide financial and technical assistance (BBNJ agreement, Article 41).

To facilitate implementation, the Agreement establishes operational elements, including a dedicated Capacity Building and Transfer of Marine Technology Committee, and also designates necessary monitoring, review, and funding mechanisms to ensure compliance and transparency (Taghizadeh and Asgarian 2024). Such mechanisms collectively reflect the sustainable equity paradigm and embody the principles of inter-/intra-generational equity and intertemporal justice. For example, Articles 42 and 43 detail the types of capacity-building and technology transfer to be prioritized, such as training programs, access to databases, and infrastructure development for marine research in developing states. Nevertheless, the way these provisions are drafted retains a degree of flexibility regarding responsibilities of the suggesting that implementation responsibilities and the effectiveness of capacity-building and technology transfer mechanisms may vary based on political will and available resources (Taghizadeh and Asgarian 2024).

As discussed in further detail in the author’s other work, more robust institutional safeguards are necessary to fully realize the objectives of inter-/intra-generational equity under the BBNJ Agreement. This includes strengthening safeguards for capacity-building, ensuring enforceable commitments to knowledge and technology transfer, and establishing clearer guidelines for benefit-sharing mechanisms (Taghizadeh 2024). Provisions such as Articles 10, 11, 42, and 53 exemplify areas where stronger and more specific obligations could be developed to ensure that the BBNJ Agreement’s implementation aligns with the overarching principles, such as the CHP, sustainable equity, the precautionary principle and the new International Economic Order supported by the developing countries (Tiller and Mendenhall 2023).

Consequently, while the BBNJ Agreement marks a transformative step toward sustainable governance of marine biodiversity in ABNJ, its long-term success will depend on how its innovative mechanisms, such as the notification system, batch identifiers, and digital clearing-house mechanisms, are operationalized. In this landscape, ensuring alignment with the principles of the common heritage of humankind, the precautionary approach, and sustainable equity will be essential to uphold the Agreement’s promise to current and future generations.

## International Community to Promote Intergenerational Responsibility Concerning Marine Biodiversity: How to Ensure Accountability towards Future Generations

The legal dilemma of how to balance the needs and rights of future generations in marine biodiversity while also addressing the immediate concerns of the present generations requires a nuanced and in-depth legal assessment. The following part of this article explores the delicate balance that must be achieved to promote intergenerational responsibility. It argues that achieving intergenerational responsibility requires a careful legal balance, which can be supported by applying the principles of the common heritage of humankind and the precautionary approach as guiding frameworks under the BBNJ Agreement. The section will then discuss the responsibilities of the international community towards future generations by explaining how to ensure inclusivity, transparency, accountability, and representation in multilateral environmental decision-making processes. The discussions will be followed by exploring the opportunities to integrate intergenerational justice and responsibility considerations into broader environmental and biodiversity governance frameworks.

### International Community’s Responsibility towards Sustainable Equity: How to Strike a Balance?

In order to achieve an effective accountability framework towards future generations, the international community must take proactive and preventive measures in shaping both international legal norms and the mechanisms through which states and other stakeholders engage in environmental decision-making. This approach can only be possible if the challenges and obstacles are thoroughly identified and addressed to ensure accountability for safeguarding marine common resources in the interest of future generations. This part of the article will explore the international community’s responsibilities to promote sustainable equity on a global scale, highlighting the critical role that accountability plays in ensuring a fair and just future for all generations.

#### Identifying Challenges and Obstacles towards Ensuring Inter-/Inter-/Intra-Generational Equity in Marine Biodiversity Governance

In the face of pressing global issues, such as climate change, resource depletion, and widening socio-economic disparities between the Global North and South,^15^ the principles of sustainable equity, particularly its intergenerational and intra-generational dimensions, have emerged as critical components of contemporary environmental law, governance, and policy-making (Ruhl and Craig 2011). Within this evolving landscape, a range of key challenges continues to hinder progress towards ensuring accountability to both present and future generations, especially where existing legal structures prove inadequate and fragmented in their implementation of these principles under national legal systems as well as international adjudication.

Among the most significant of these challenges is the insufficiency of the current international accountability framework to comprehensively address inter-/intra-generational equity within marine biodiversity governance. From one perspective, current legal frameworks are mainly designed to focus on the present over-exploitation of biological resources, often overlooking the broader implications for future generations’ rights and ecological well-being. To put it differently, they often prioritize immediate economic development over the long-term sustainability of marine ecosystems, often lacking mechanisms that adequately incorporate future-oriented impact assessments or enforce accountability for environmental harm (Eisenmenger et al. 2020).

Additionally, from a strict legal standpoint, the absence of clarity and international consensus on the precise scope and content of inter-/intra-generational equity presents another major obstacle. Not only is there limited legal recognition of the rights of future generations to a healthy environment and sustainable resources management (Magraw and Siemes 2023), but there is also no universally agreed-upon definition or operational framework for applying inter-/intra-generational equity in policy and practice. This ambiguity hampers the formulation of concrete legal measures and renders it increasingly complicated to assert claims on behalf of future generations, given the lack of legal standing or procedural mechanisms available to support such actions.

The critical challenge lies not only in articulating the equity principles under the existing international and national legal frameworks but also in overcoming the substantial obstacles hindering their operationalization and enforcement. Compounding this issue is the disconnect between the aspirational language found in international environmental instruments and the enforceability of those commitments. Although numerous international environmental instruments, as previously outlined, explicitly reference the importance of sustainable equity and intergenerational responsibility, they frequently fall short in establishing binding obligations or enforceable mechanisms to hold states accountable for their harmful activities. As a result, a significant gap persists between the normative aspirations of the international community and their translation into enforceable legal standards.

Moreover, from a pragmatic perspective, the existing power imbalances between developed and developing countries from the Global North and South further complicate efforts to achieve equitable governance of marine biodiversity and foster global cooperation (Campbell 2022). While industrialized nations, historically as major contributors to environmental degradation, often evade their full responsibility (Gabbatiss 2024), developing and least developed countries face intense pressures to exploit their natural resources for immediate economic gain, even at the expense of long-term sustainability (World Commission on Environment and Development 1987). Without a robust regulatory framework governing equitable resources distribution in ABNJ, alongside an articulated interpretation of inter-/intra-generational equity, developing nations continue to risk marginalization and may be excluded from meaningful participation in scientific research into MGRs and from fair access to the benefits derived from such resources (Asimakopoulou and Mohammad 2019).

The lack of rigorous enforcement of international law addressing sustainable equity paradigms, coupled with insufficiently stringent legal penalties, often creates significant judicial and institutional barriers to realizing intergenerational justice (Bertram 2023). This challenge is further compounded by the fact that multiple jurisdictions adopt diversified legal approaches to environmental preservation, biological resource management, and intergenerational justice, which leads to a fragmented legal landscape that complicates the consistent enforcement of intergenerational accountability (Sugirman 2023). In particular, some jurisdictions lack the institutional capacity or legal mechanisms to recognize and adjudicate claims concerning intergenerational harms or inequities, thereby weakening the protection afforded to future generations.

Moreover, international courts and tribunals may be constrained by jurisdictional limitations, which not only delay justice for communities affected by environmental degradation and socio-economic inequity but also diminish the effectiveness of international redress mechanisms. As a result, such fragmentation across legal and institutional frameworks leads to the inconsistent application of regulations governing human activities in ABNJ, while simultaneously hindering global coordination and coherence among states, regional and global competent organisations, and all relevant stakeholders (Gjerde 2018). Accordingly, addressing these issues demands both a refinement of international legal standards and a rebalancing of global governance structures to ensure more inclusive, just, and sustainable stewardship of marine biodiversity for both present and future generations.

#### Intergenerational Responsibility towards Marine Biodiversity as Common Heritage of Humankind

The evolving body of international environmental law recognizes that states are not only accountable towards their present populations but also bound by their responsibilities to preserve the global environment for the benefit of future generations (Agius 1988). States, as the main “stewards” of the ocean environment, have a profound obligation to protect biological ecosystems and promote sustainable development, not only for the benefit of the present generation but with the foresight to ensure the sustainability of resources for generations yet unborn (Mendenhall and Tiller 2023). However, the existing international legal frameworks often fail to adequately account for the rights of future generations and the need for proactive, consolidated action to protect the environment. As stated earlier, states usually prefer to prevail in 'presentism'—the tendency to prioritize the interests of the current generation and overlook the needs and rights of future generations, rather than taking approaches toward achieving inter-/intra-generational equity (Pedersen and Sulyok 2024).

The fundamental grounds for incorporating futuristic, forward-looking obligations of environmental sustainability into international law lie in the recognition of the common heritage of humankind principle and the intrinsic value of shared natural resources, including biological diversity, and the marine environment (United Nations General Assembly 1982). These rudimentary obligations, though still evolving, are unique in nature, transcending national jurisdictions and encompassing duties to uphold global values and universal interests, such as human rights and environmental integrity (Urs 2021). Within the framework of emerging international legal instruments, particularly the BBNJ Agreement, such obligations are operationalized through collective governance mechanisms. Accordingly, each member party to these international mechanisms shares both common and differentiated responsibilities as well as a legitimate interest and potentially legal standing in addressing instances of non-compliance by other parties within these treaty mechanisms (Birnie et al. 2009).

At the heart of a critical examination of how existing legal frameworks can be strengthened to uphold the rights of current and future generations lies the interplay of intergenerational responsibility frameworks and the common heritage of humankind concept, inquiring how these regulatory frameworks can provide essential tools for fostering inter-/intra-generational equity. The common heritage of humankind principle emerges as a pragmatic solution towards operationalizing peremptory obligations within the context of inter-/intra-generational justice. This principle operates on the starting premise that international cooperation and shared responsibility among all stakeholders, including states, civil society, and indigenous peoples, are essential elements for the sustainable, equitable management of global commons, ranging from climate stability to biodiversity conservation.

From a strictly legal perspective, the ICJ has established that certain obligations, such as the prohibition of genocide, racism, and ecological harm, are owed to all, ingrained in the common values of the international community, which compels states to act in a manner that upholds these values even when the harm is indirect (Crawford 2001; Kawasaki 2006). As highlighted by the ICJ in its Barcelona Traction case, these obligations may fall under the description of superior values of the international community defined as “*obligations erga omnes*” or “*obligations owed to the international community as a whole*.” (ICJ, *Barcelona Traction, Light and Power Company*, Judgment 1970, Para 33).

The growing recognition of the irreversible value of marine biodiversity to the global community, taking into account the significant developments in international legal instruments addressing environmental protection, would suggest that the obligation to protect marine biological diversity may be construed as an “*erga omnes* obligation”, by the futuristic obligations of environmental sustainability law (Alarcon and Tigre 2023). Some scholars suggest that the “starting premise is that BBNJ obligations will be owed to the international community as a whole: *erga omnes* obligations.” Such characterization in his idea “is compelled by the agreement’s intended scope and purpose”. (Payne 2022, 24-26).

In this landscape, *erga omnes* obligations represent a fundamental tenet of international law (Memeti, and Nuhija 2013), referring to obligations that are owed to the international community as a whole, rather than to an individual state or groups of states, regardless of their direct interest in the governance of genetic resources (Shchokin 2021). The recognition of *erga omnes* characteristics for inter-/intra-generational equity asymmetries in the context of ecological biodiversity provides a robust legal framework that reinforces the necessity for current generations to protect and adequately preserve the shared resources of our common environment for the benefit of future inhabitants.

The dual framework of *erga omnes* obligations and the common heritage of humankind principle and their intersection presents a unique opportunity to institutionalize intergenerational responsibility in global biodiversity governance (Taghizadeh 2024). For instance, international agreements on biodiversity conservation must explicitly reference *erga omnes* obligations to mitigate harm to the environment, thereby linking current legislative frameworks with the moral imperative of preserving ecological integrity for future generations. The common heritage of humankind principle facilitates this by encouraging joint initiatives among states and all other stakeholders to create adaptive management plans for global resource systems that reflect the foresight required in environmental stewardship (Stern 2011).

The core premise of this article is not only the necessity of embedding binding obligations towards future generations into the fabric of international law, but also the recognition that the BBNJ Agreement provides a critical platform for advancing this objective. By operationalizing inter-/intra-generational equity through its collective governance mechanisms, the BBNJ Agreement exemplifies how international treaties can move beyond aspirational commitments toward concrete legal frameworks that promote sustainability and shared responsibility (Goldmann 2012). This approach entails an obligation for the international community to move towards a more responsible and inclusive model for global resource governance, thereby addressing existing gaps in accountability and intergenerational responsibility (Hernández Guzmán and Hernández García de Velazco 2023). Consequently, as we confront the complexities of contemporary environmental challenges of our time, establishing such legal infrastructures, anchored in futuristic principles such as common heritage of humankind and *erga omnes* obligations, will be pivotal in securing a sustainable and equitable future for all generations.

### Redefining Accountability Frameworks and Inter-/Intra-generational Justice: Ensuring Inclusivity, Diversity, and Representation in Multilateral Policy-Making Processes

The current state of environmental resource management governance, particularly with regard to marine biodiversity, remains insufficiently robust, with notable gaps in accountability mechanisms and inadequate integration of key stakeholders, including indigenous peoples, traditional knowledge holders, and marginalized communities.^16^ This article explores how to strengthen international accountability frameworks by focusing on two crucial aspects: first, ensuring inclusivity and representation in decision-making, particularly through the recognition of indigenous intergenerational knowledge and the active involvement of local communities; and second: integrating inter-/intra-generational equity asymmetries into broader environmental and biodiversity governance frameworks, including proposals for strengthening the integration of inter-/intra-generational equity in the BBNJ Agreement.

#### Ensuring Accountability towards Indigenous Peoples and Local Communities: Promoting Intergenerational Ecological Knowledge of the Marine Environment

Indigenous peoples and local communities (IPLCs) have long been the spiritual custodians of traditional ecological knowledge systems, particularly in relation to marine ecosystems and sustainable resource management (Diaz et al. 2019). Most of the indigenous communities rely on customary management systems of biological resources for their livelihoods, and the conservation and sustainable use of these resources are crucial for their continued existence (UNESCO-IOC & UNESCO-LINKS 2024). The rapid pace of biodiversity loss and environmental degradation deeply threatens the sustainable future and cultural identities of these indigenous and local communities and they remain susceptible to further dispossession and exploitation of the resources (Dawson et al. 2021).

The preservation of traditional knowledge is fundamental to safeguarding the cultural heritage and intellectual property rights of Indigenous Peoples and Local Communities (IPLCs), while also supporting the continued transmission of their cultural practices (Keele 2024). Both the Nagoya Protocol and the BBNJ Agreement recognize the requirement of obtaining the free, prior, and informed consent (FPIC) of Indigenous Peoples and local communities as a condition for benefit-sharing related to traditional knowledge associated with genetic resources (Nagoya Protocol, Arts 7, 16; BBNJ Agreement, Art 13). Several sub-principles emerge from this overarching commitment, including the need for meaningful consultation with traditional knowledge holders in the development of proposals, securing their FPIC, ensuring respectful and appropriate handling of traditional knowledge, and promoting the fair and equitable sharing of benefits arising from digital sequence information related to such knowledge (Friedman 2025).

The following part explores the legal measures necessary to effectively promote intergenerational knowledge systems of the marine environment, while also addressing existing gaps in environmental law and management practices. One such gap surrounds an inadvertent lack of understanding of the rights and aspirations of Indigenous Peoples and local communities (IPLCs) prior to the engagement process, which often results in superficial participatory frameworks that fail to genuinely include them in environmental decision-making processes (UNESCO-IOC &UNESCO-LINKS 2024). To advance meaningful inclusion and authentic participation of IPLCs, it is imperative to move beyond past tokenistic consultations and instead foster engagement processes that authentically reflect the rudimentary values and priorities embedded in their traditional ecological knowledge systems (Gordon et al. 2020).

Significantly, what is highly controversial is that the existing legal frameworks, whether nationally or internationally, do not adequately address the perspectives and interests of indigenous peoples and future generations; thus, these communities’ interests may be sidelined in favor of more immediate legal concerns (Zurba et al. 2024). This gap often leads to the regulation of laws and policies that fail to address the long-term implications for environmental justice and sustainable equity. Besides, from a stricter legal point of view, failure to distinguish between stakeholders and rights-holders in some policy arenas results in homogenisation of participants in decision making processes. (UNESCO-IOC &UNESCO-LINKS 2024).

Furthermore, multi-stakeholder platforms that include civil society, indigenous communities, and marginalized groups' participation in multilateral decision-making processes, such as the BBNJ process, foster a greater understanding of the interconnectedness of equity and sustainability (Strand et al. 2022). Such transformative platforms towards integrating indigenous peoples and local communities’ ecological knowledge into ocean science and management require a considerable shift towards more sustainable and equitable practices, taking into account the needs and aspirations of all stakeholders, including traditional knowledge right holders (Caldeira et al. 2025). These decisive approaches can ultimately lead to holding states and other subjects of international law accountable for their commitments towards both present and future populations of the IPLCs.

The BBNJ Agreement’s formal recognition of the rights of Indigenous Peoples and Local Communities (IPLCs) over their traditional knowledge and genetic resources represents a significant advancement, as it seeks to guarantee these communities equitable access to benefits derived from their cultural heritage, thereby contributing to the enhanced protection and sustained preservation of their traditional knowledge systems. While the BBNJ Agreement acknowledges the distinct rights and special circumstances of IPLCs, Small Island Developing States, and landlocked developing countries, particularly emphasizing their entitlement to uphold and maintain traditional knowledge associated with genetic resources,^17^ it falls short in explicitly establishing detailed guidelines and frameworks for sustainable harvesting and utilization practices. Such frameworks are essential to ensure that traditional ecological knowledge is respected and that these practices remain viable and resilient across generations.

A critical challenge moving forward will be the effective operationalization of these provisions in practice, as without careful implementation, there is a risk of perpetuating superficial or tokenistic forms of participation that do not fully empower IPLCs. Consequently, adopting a comprehensive and integrative approach will be indispensable to ensure the meaningful inclusion of all relevant stakeholders, including non-state actors, non-governmental organizations (NGOs), and principally IPLCs, within ocean governance structures and related follow-up regimes (Taghizadeh 2024). Only through such genuine and participatory engagement can the BBNJ framework realize its full potential to uphold the rights and aspirations of traditional knowledge holders in a manner that is both substantive and enduring across all generations.

#### Integrating Inter-/Intra-Generational Equity Asymmetries into Broader Multilateral Biodiversity and Environmental Governance Frameworks

As projected by this article, effective environmental protection requires not only addressing contemporary challenges but also embracing a forward-looking approach that secures the resilience and sustainability of ecosystems for the benefit of future generations. A fundamental aspect of this approach involves a critical re-evaluation of global governance structures, aimed at embedding ethical and legal responsibilities towards future generations within international law (Weiss 1990). This involves safeguarding the rights and interests of not only current stakeholders but also indigenous peoples and local communities (IPLCs) and future generations, necessitating transparent, accountable, and inclusive decision-making processes. The existing lacuna in international law highlights the urgent need for mechanisms that bridge these intergenerational considerations, intertwining them with the contemporary governance frameworks.

The BBNJ Agreement embodies a critical platform to align international legal frameworks with the principles of inter-/intra-generational equity and pave the way to achieve a genuine commitment of the whole international community towards a more sustainable, responsible, and resilient future. One significant strategy for enhancing the integration of equity principles into broader multilateral contexts involves establishing strategic connections between the BBNJ Agreement and other pertinent international mechanisms, especially those focused on marine genetic resources and environmental protection.^18^ This alignment can facilitate a cohesive set of objectives and targets, enabling a holistic approach that effectively addresses inter-/intra-generational equity issues at both global and national levels, thereby filling the existing lacuna in coherence across various legal frameworks.

Additionally, advancing inter-/intra-generational equity paradigms necessitates the development of enforceable normative guidelines to ensure responsible and sustainable resource management, including concrete implementing measures such as limiting marine resource exploitation and designating marine biodiversity protected areas (MPAs) (Article 19.5 and Annex I of the BBNJ Agreement), supported by robust and effective monitoring and enforcement mechanisms (Liu 2024). While the BBNJ Agreement aspires to reconcile the legitimate needs and interests of both present and future generations, its potential effectiveness could be enhanced by the inclusion of clear, detailed guidelines to prioritize these interests. Such guidelines might be appropriately incorporated at a later stage, for instance, as an annex to the Agreement, providing flexibility for iterative refinement and facilitating stronger implementation over time.

As previously discussed in this article, the immediate economic interests of current generations frequently conflict with the long-term sustainability asymmetries in the interest of future generations (Morgera (2024)). This tension is especially pronounced in the context of marine biological resources, where the economic appeal of immediate exploitation frequently overshadows the long-term irreversible environmental damages that result from overutilization and habitat degradation, further highlighting the existing lacuna in compliance mechanisms (Worm et al. 2019). Moreover, incorporating liability and compensation frameworks into the BBNJ Agreement could form a critical advance towards ensuring effective protections for the marine environment, as it holds states accountable for harm inflicted by their activities on marine ecosystems beyond national jurisdiction (Mendenhall and Tiller 2023). The Agreement includes the Polluter-Pays principle in Article 7(a), which requires states to attribute liability and costs to those responsible for pollution in areas beyond national jurisdiction, thereby incentivizing responsible behavior and addressing transboundary harm (Article 7(a) of the BBNJ Agreement). However, the implementation of such frameworks presents both challenges and opportunities.

One significant challenge is the need for international cooperation to enforce liability and compensation mechanisms, given the transnational nature of marine pollution and harm. In this landscape, the current absence of standardized metrics for measuring equity, combined with disparities in data availability and quality, impedes efforts to assess compliance with international obligations.^19^ For instance, the absence of clear indicators to quantify inter-/intra-generational equity in national reporting processes poses significant challenges to transparency and accountability, particularly concerning the protection of traditional ecological knowledge as well as local and native scientific contributions (Sinthumule 2023; Shackeroff and Campbell 2007).

To tackle these challenges, it is essential to ensure that standardized metrics and data management practices are developed within a robust accountability framework to enable transparent evaluation of liability claims and compensation processes and their policy impacts. Within this framework, specific attention must be paid to how state reporting and compliance committees could integrate qualitative and quantitative indicators to evaluate progress toward equity commitments. These metrics should capture equity considerations for both current and future generations, ensuring that the benefits and burdens of marine resource use are distributed fairly across generations. Additionally, integrating traditional knowledge into liability and compensation frameworks to be established by the future regulatory frameworks under the BBNJ Agreement can support restorative justice for affected communities and ecosystems, fostering a more equitable and sustainable approach to marine conservation.

A practical recommendation for fortifying the principle of inter-/intra-generational equity in the context of the BBNJ Agreement is the establishment of a dedicated working group or committee focused extensively on intergenerational justice and responsibility. This could be established as a new subsidiary body under the Conference of the Parties (COP), as authorized by Article 47 of the Agreement, or as a specialized working group under the Implementation and Compliance Committee (Article 55), which facilitates compliance with the Agreement’s principles, including inter-/intra-generational equity. Such a body would facilitate the exchange of best practices among states, stakeholders, and experts to effectively integrate inter-/intra-generational equity asymmetries into marine biodiversity management decision-making processes (Boiral and Heras-Saizarbitoria 2017). Additionally, creating an *Inter-/Intra-generational Equity Advisory Body* within the BBNJ governance structure could further embed long-term perspectives into decision-making, potentially involving representation from youth, indigenous peoples, and local communities as well as advocates for future generations. This advisory body could serve as a critical mechanism to address existing gaps in representation, inclusivity, and advocacy for posterity rights, ensuring that the voices of these marginalized communities are appropriately prioritized.

Moving forward, to solidify the existing lacuna in international legal framework towards acknowledging and actualizing inter-/intra-generational justice, the BBNJ Agreement can establish a *sui generis* framework that integrates the precautionary approach, global commons principles and sustainable equity paradigms, specifically tailored to ensure conservation and sustainable use of marine biological resources for future generations. This framework is made possible by leveraging the Agreement’s existing provisions, such as Article 2, which emphasizes conservation “for the present and in the long term,” inherently recognizing the need to protect marine biodiversity for future generations. Article 7 incorporates the precautionary approach, requiring decision-makers to act cautiously when activities risk long-term harm to the marine environment, and the principle of equity, which can encompass intergenerational fairness. For instance, EIAs under Part IV can be designed to explicitly evaluate long-term impacts on marine ecosystems, ensuring that activities such as deep-sea mining or overfishing do not compromise future generations’ access to a healthy ocean.

Additionally, Part II’s provisions on MGRs enable fair and equitable benefit-sharing, which could include mechanisms to allocate benefits toward long-term conservation or funds for future generations. The Conference of the Parties, established under Article 47, has the authority to develop guidelines or establish a dedicated working group on inter-/intra-generational equity, potentially involving youth and indigenous peoples to ensure inclusive representation of future generations and holders of traditional ecological knowledge. By embedding these mechanisms within its implementation, the BBNJ Agreement can create a unique framework that prioritizes the rights of future generations to a healthy and sustainable marine environment through inter-/intra-generational equity, guiding decision-makers to safeguard the marine commons for the benefit of all humanity, present and future.^20^

## Conclusion

Sustainable equity paradigms, encompassing both the evolutionary concept of intergenerational equity (fairness to future generations) and intra-generational equity (the equitable distribution of resources and opportunities among current populations), are fundamental to contemporary environmental governance. While intergenerational equity emphasizes the responsibility of present generations to safeguard the rights and needs of those yet to come, intra-generational equity demands attention to the disparities and justice concerns among existing communities, particularly marginalized and vulnerable groups. These principles have gained significant prominence in international environmental law discourse in recent years, especially in the Anthropocene era (Weiss 2019), characterized by unprecedented technological development and environmental pressures. This dual focus underscores that equity is not merely an ethical aspiration but a binding legal obligation that reinforces shared responsibilities across temporal and societal divides in global governance and resource management.

In light of the critical challenges facing the marine ecosystems and the irreparable nature of their biodiversity and genetic resources, it is highly imperative to adopt a multi-faceted approach that integrates inter-/intra-generational equity perspectives in environmental decision-making. Achieving this requires legal reforms and strengthened enforcement mechanisms that reflect a fundamental paradigm shift in how international law conceptualizes responsibility, liability, and accountability, not only toward future generations but also within present societies. The evolving body of this research advocates for a profound transformation in international legal frameworks to embrace a proactive, forward-looking orientation, anticipating the needs and priorities of future populations, while simultaneously addressing existing inequities among current stakeholders. This comprehensive governance approach should interweave core principles such as the common heritage of humankind, the precautionary approach, and shared inter-/intra-generational responsibilities, thereby fostering a holistic stewardship of marine biodiversity moving beyond mere compliance with existing legal frameworks.

Within this evolving legal landscape, the BBNJ Agreement emerges as a transformative platform poised to rectify both inter-/intra-generational equity asymmetries. By embedding these principles into broader multilateral governance frameworks, the Agreement promotes the sustainability and resilience of marine biodiversity for the benefit of all generations. The pivotal role of the BBNJ Agreement in advancing the futuristic revolutionary principles of international environmental law is exemplified through its principles and provisions, reinforcing shared intergenerational responsibility of the international community to safeguard the ecological integrity of oceans and their marine genetic resources, through principles such as the common heritage of humankind. The evolving *sui generis* legal framework concerning intergenerational responsibility proposed in this article aims to fill the existing lacunae by establishing a shared responsibility towards marine resources, recognizing these resources as communal heritage belonging to all humanity.

Ultimately, enhancing international cooperation and collaboration on multilateral platforms, along with strengthening management strategies and public-private partnerships at the global level, through acknowledging the shared responsibility for ocean stewardship, is the last piece of the puzzle to strike a balance between the needs of both current and future generations. In this context, the coordinated exchange of best practices, scientific knowledge, and inclusive engagement with IPLCs by the international community are critical for addressing common challenges and operationalizing equity principles. Such collaborative efforts towards the integration of inter-/intra-generational equity within broader environmental governance frameworks contribute to advancing environmental management across theory, policy, and practice, thus fostering a more just, equitable, and sustainable future for all humanity.

## Data Availability

No datasets were generated or analysed during the current study.

## Footnotes

1 Sustainable development, as conceptualized by the Brundtland Report, refers to “development that meets the needs of the present without compromising the ability of future generations to meet their own needs”. Schlosberg, D., & Collins, L.B. 2014. From environmental to climate justice: Climate change and the discourse of environmental justice. *WIREs Climate Change*, *5(3)*, 359–374, 10.1002/wcc.275 available at, https://www.ipbes.net/node/41377 (Accessed: 5 August 2025).

2 The UNCLOS utilizes the term ‘Common Heritage of Mankind’ (CHM), whereas the BBNJ uses ‘Common Heritage of Humankind Principle’ (CHP) which seems to serve as a more gender neutral term and will be utilised in this article more often.

3 According to the Max Planck Encyclopedias of International Law: “The precautionary principle (approach) is part of a system of rules designed to guide the decision-making of States towards the goal of an environmentally sustainable development. It has become a guiding principle of modern international law and, increasingly, of national instruments and jurisprudence relating to protection of the environment and conservation of natural resources.”, Meinhard Schröder .^2014^. ‘Precautionary Approach/Principle’, Max Planck Encyclopedias of International Law, Available at, https://opil.ouplaw.com/display/10.1093/law:epil/9780199231690/law-9780199231690-e1603 (Accessed: 5 August 2025).

4 To study moral or ethical perspectives and more generally planetary governance issues, see Young, O. R. (2021). Grand challenges of planetary governance: global order in turbulent times.’ In *Grand Challenges of Planetary Governance*. (pp 1-192).

5 UNFCCC. (1992). United Nations Framework Convention on Climate Change (in force March 21, 1994). Art. 3(1). https://unfccc.int. Article 3.1 of the UNFCCC states that Parties should “protect the climate system for the benefit of present and future generations of humankind, on the basis of equity and in accordance with their common but differentiated responsibilities and respective capabilities. Accordingly, the developed country Parties should take the lead in combating climate change and the adverse effects thereof”.

6 Article 7 (e) of the BBNJ Agreement refers to “The precautionary principle or precautionary approach, as appropriate”, without further explanation as to what could be considered “as appropriate” or the distinction between “precautionary principle” and “precautionary approach.” For a further examination, see Gottlieb. et al. (2025) 102.

7 “The preventive and precautionary principles are landmark principles of international environmental law. A dividing line could be drawn between these two principles: prevention is addressing tangible risks whilst precaution deals with scientific uncertainty.” Sadeleer, N. de. (2010). The principles of prevention and precaution in international law: two heads of the same coin?, *Research Handbook on International Environmental Law* (pp 183-199). http://tradevenvironment.eu/wp-content/uploads/2021/10/Research-Handbook-of-IEL.pdf

8 “In order to protect the environment, the precautionary approach shall be widely applied by States according to their capabilities.“ See Principle 15 of the Rio Declaration on Environment and Development adopted at the UN Conference on Environment and Development, 1992, UN Doc. A/CONF.151/26, reprinted in 31 ILM 874 (1992). http://www.un.org/documents/ga/conf151/aconf15126-1annex1.htm.

9 For further study, See Tickner, J. (2003). *The Precautionary Principle in Action, A handbook* (First ed.). Science and Environmental Health Network, http://calepa.ca.gov/wp-content/uploads/sites/6/2016/10/EnvJustice-Documents-2003yr-Appendices-AppendixI.pdf

10 Gene technology involves modifying organisms by incorporating, deleting or altering one or more genes or genetic sequences to introduce or alter a specific characteristic(s). For further study, See Weier, A., & Lok, P. (2007). Precaution and the Precautionary Principle: two Australian case studies. *Productivity Commission Staff Working Paper*, 47 Melbourne.

11 See the history of these contentious debates in the intergovernmental conferences (IGC) meetings prior to the adoption of the BBNJ Agreement: Tiller, R., Mendenhall, E., De Santo, E., and Nyman, E. (2023). Shake it off: Negotiations suspended, but hope simmering, after a lack of consensus at the fifth intergovernmental conference on biodiversity beyond national jurisdiction. *Marine Policy* 148: 105457. 10.1016/j.marpol.2022.105457. Also See Mendenhall, E., De Santo, E., Jankila, M., Nyman, E., and Tiller, R. (2022) Direction not detail: Progress towards consensus at the fourth intergovernmental conference on biodiversity beyond national jurisdiction. *Marine Policy* 146: 105309. 10.1016/j.marpool.2022.105309

12 Article 1.8 of the BBNJ Agreement outlines a governance framework for MGRs, defined as “any material of marine plant, animal, microbial or other origin containing functional units of heredity of actual or potential value”.

13 This can be Inferred from the guiding principle of “common heritage of humankind” and its potential implications for future generations.

14 This differs significantly from the benefit-sharing of the Nagoya Protocol under the CBD, where monetary benefits are shared bilaterally, on mutually agreed terms, but are not necessarily used towards advancing the objectives of the CBD.

15 The Global North generally refers to developed countries that are characterized by high levels of economic development, industrialization, well-developed infrastructure, technical advancement and macroeconomic and political stability. The developing countries of the Global North, on the other hand, are characterized by lower levels of economic development, political stability and technical infrastructures. For further detailed study, see Kowalski, A. (2020). Global South-Global North Differences. ResearchGate. Springer Nature Switzerland AG. https://www.researchgate.net/publication/342507407

16 For a more detailed study on social and intergenerational equity and its human effects, see: Summers J.K, Smith L.M. 2014. The role of social and intergenerational equity in making changes in human well-being sustainable. *Ambio*, *43*(6):718-28. 10.1007/s13280-013-0483-6.

17 BBNJ Agreement, e.g., Preamble and Articles 7, 9, 11, 13. “The BBNJ Agreement also allows the exclusion of traditional knowledge concerning marine genetic resources of Indigenous Peoples and local communities, many of which are part of developing states, from the clearing-house mechanism.”, Friedman, (2025) 7.

18 These frameworks, inter alia include UNCLOS, the recent WIPO Treaty on Intellectual Property, Genetic Resources and Associated Traditional Knowledge (GR-ATK Treaty), the UNFCCC, the CBD and the Nagoya Protocol.

19 For an understanding of metrics in measuring inequality, see Custodio, H.M., Hadjikakou, M., Bryan, B. A. (2023) A review of socioeconomic indicators of sustainability and wellbeing building on the social foundations framework, *Ecological Economics*, 203, 107608), 2–4. 10.1016/j.ecolecon.2023.107608.

20 The BBNJ supports the protection of every one’s human right to a healthy environment. Bennet, N., Morgera, E., & Boyd, D. (2024). The human right to a clean, healthy, and sustainable ocean. *NPJ Ocean Sustainability, 3*, 19.
